# Reduction of *BMPR2* mRNA Expression in Peripheral Blood of Pulmonary Arterial Hypertension Patients: A Marker for Disease Severity?

**DOI:** 10.3390/genes13050759

**Published:** 2022-04-26

**Authors:** Vivienne Theobald, Nicola Benjamin, Hans-Jürgen Seyfarth, Michael Halank, Marc A. Schneider, Sarah Richtmann, Katrin Hinderhofer, Panagiota Xanthouli, Benjamin Egenlauf, Rebekka Seeger, Marius M. Hoeper, Danny Jonigk, Ekkehard Grünig, Christina A. Eichstaedt

**Affiliations:** 1 Center for Pulmonary Hypertension, Thoraxklinik Heidelberg gGmbH at Heidelberg University Hospital, 69126 Heidelberg, Germany; v.theobald@stud.uni-heidelberg.de (V.T.); Nicola.Benjamin@med.uni-heidelberg.de (N.B.); Panagiota.Xanthouli@med.uni-heidelberg.de (P.X.); Benjamin.Egenlauf@med.uni-heidelberg.de (B.E.); Rebekka.Seeger@med.uni-heidelberg.de (R.S.); Ekkehard.Gruenig@med.uni-heidelberg.de (E.G.); 2 Translational Lung Research Center Heidelberg (TLRC), German Center for Lung Research (DZL), 69120 Heidelberg, Germany; marc.schneider@med.uni-heidelberg.de (M.A.S.); sarah.richtmann@med.uni-heidelberg.de (S.R.); 3 Department of Pneumology, Medical Clinic II, University Hospital of Leipzig, 04103 Leipzig, Germany; Hans-Juergen.Seyfarth@medizin.uni-leipzig.de; 4 Medical Clinic I, University Hospital of Dresden, 01307 Dresden, Germany; Michael.Halank@uniklinikum-dresden.de; 5 Translational Research Unit, Thoraxklinik Heidelberg gGmbH at Heidelberg University Hospital, 69126 Heidelberg, Germany; 6 Division of Systems Biology of Signal Transduction, German Cancer Research Center (DKFZ), 69120 Heidelberg, Germany; 7 Laboratory for Molecular Diagnostics, Institute of Human Genetics, Heidelberg University, 69120 Heidelberg, Germany; Katrin.Hinderhofer@med.uni-heidelberg.de; 8 Clinic for Pneumology, Hannover Medical School, Biomedical Research in End-Stage and Obstructive Lung Disease Hannover (BREATH), German Center for Lung Research (DZL), 30625 Hannover, Germany; Hoeper.Marius@mh-hannover.de; 9 Institute for Pathology, Hannover Medical School, Biomedical Research in End-Stage and Obstructive Lung Disease Hannover (BREATH), German Center for Lung Research (DZL), 30625 Hannover, Germany; Jonigk.Danny@mh-hannover.de

**Keywords:** *BMPR2* pathogenic variant, *BMPR2* mRNA expression, pulmonary arterial hypertension, heritable pulmonary arterial hypertension

## Abstract

Pulmonary arterial hypertension (PAH) can be caused by pathogenic variants in the gene bone morphogenetic protein receptor 2 (*BMPR2*). While *BMPR2* protein expression levels are known to be reduced in the lung tissue of heritable PAH (HPAH) patients, a systematic study evaluating expression in more easily accessible blood samples and its clinical relevance is lacking. Thus, we analyzed the *BMPR2* mRNA expression in idiopathic/HPAH patients and healthy controls in blood by quantitative polymerase chain reaction and protein expression by enzyme-linked immunosorbent assay. Clinical parameters included right heart catherization, echocardiography, six-minute walking test and laboratory tests. *BMPR2* variant-carriers (n = 23) showed significantly lower *BMPR2* mRNA expression in comparison to non-carriers (n = 56) and healthy controls (n = 30; *p* < 0.0001). No difference in *BMPR2* protein expression was detected. Lower *BMPR2* mRNA expression correlated significantly with greater systolic pulmonary artery pressure and pulmonary vascular resistance. Higher *BMPR2* mRNA expression correlated with greater glomerular filtration rate, cardiac index and six-minute walking distance. We demonstrated the feasibility to assess *BMPR2* expression in blood and, for the first time, that *BMPR2* mRNA expression levels are significantly reduced in variant carriers and correlated with clinical parameters. Further studies may evaluate the usefulness of *BMPR2* mRNA expression in blood as a new marker for disease severity.

## 1. Introduction

Pulmonary arterial hypertension (PAH) is characterized by a progressive pulmonary vascular remodeling which leads to increased pulmonary vascular resistance, right ventricular hypertrophy and, ultimately, right heart failure [1]. As of today, different pathogenic germline variants in 18 genes have been described in heritable PAH (HPAH), of which new ones are constantly being discovered [2]. Most of these pathogenic variants affect the bone morphogenetic protein receptor type II (*BMPR2*) gene and are identified in 50–80% of HPAH patients and in about 15–20% of idiopathic PAH (IPAH) patients [3,4]. These disease-causing variants are inherited in an autosomal dominant manner, but with reduced penetrance. Thus, not all family members carrying a pathogenic variant develop manifest PAH. Reasons for this reduced penetrance may be the higher expression of the wildtype variant in a heterozygous carrier or the need for additional disease triggers, such as another pathogenic variant as a “second hit” in affected patients [5].

In healthy lung tissue, *BMPR2* is mainly expressed in endothelial cells [6]. In PAH patients, *BMPR2* expression could also be detected in the endothelium and the myofibroblasts of plexiform lesions [7]. However, the *BMPR2* protein expression was greatly reduced in pulmonary endothelial cells of *BMPR2* variant carriers [6]. A less pronounced but still highly significant reduction in expression was identified in non-variant carriers in comparison to healthy controls [6].

In line with *BMPR2* variants disrupting important molecular pathways, disease severity has been shown to be significantly worse in variant carriers [4,8]. HPAH patients with pathogenic variants in the *BMPR2* gene were characterized by an earlier age at diagnosis, worse hemodynamics and higher rate of lung transplantation as well as all-cause mortality compared to non-variant carriers [4,8]. Hence, it is important to regularly screen apparently healthy *BMPR2* variant carriers clinically to identify possible disease onset as early as possible [9,10]. An early detection allows to start treatment at an early stage and may lead to a better outcome [9]. Since lung biopsies are contraindicated in PAH patients due to a high risk of morbidity and mortality [1], our objective was, firstly, to clarify whether *BMPR2* mRNA and protein blood expression levels showed differences in affected *BMPR2* variant carriers in comparison to non-variant carriers and to healthy controls; and secondly, we aimed to correlate *BMPR2* mRNA and protein blood expression levels with clinical and laboratory parameters. 

## 2. Materials and Methods

### 2.1. Study Design

This was a prospective, explorative, cross-sectional study investigating the RNA and protein expression level of *BMPR2* in the blood of PAH patients and healthy controls. The majority of patients was diagnosed and treated at the Centre for Pulmonary Hypertension, Thoraxklinik Heidelberg gGmbH at Heidelberg University Hospital, Germany. Three of the *BMPR2* variant carriers were treated at the Carl Gustav Carus University Hospital Dresden, Germany and four at the University Hospital Leipzig, Germany. Age- and gender-matched healthy controls were invited to participate if a routine blood draw was performed. 

### 2.2. Clinical Investigations

Clinical parameters for patient characterization were obtained from clinical records at the time of study enrollment and included demographic data, comorbidities, vital signs, hemodynamics measured by echocardiography and right heart catheterization, spirometry, blood gas analysis, lung function, 6 min walking distance, as well as the laboratory parameters for the assessment of renal function (creatinine, urea, and glomerular filtration rate) and other routine parameters, such as N-terminal pro-brain natriuretic peptide (NT-proBNP). If no echocardiography, right heart catheterization, spirometry or lung function was performed during the inclusion visit, the data from the most recent examination were used.

### 2.3. Genetic Investigations

DNA was extracted from 3–10 mL of EDTA-blood samples using an automated procedure (Autopure or QIAsymphony, Qiagen, Hilden, Germany). Genetic diagnostic analysis was carried out with our patented (EP3507380) PAH-specific gene panel, including the following PAH diagnostic genes: *ACVRL1*, *AQP1*, *ATP13A3*, *BMPR1B*, *BMPR2*, *CAV1*, *EIF2AK4*, *ENG*, *GDF2*, *KCNA5*, *KCNK3*, *KLF2*, *SMAD4*, *SMAD9*, *SOX17* and *TBX4* (customized SureSelect QXT kit, Agilent, Germany). The procedure was carried out as previously described [2,11]. 

Multiplex ligation-dependent probe amplification (MLPA) was performed in addition to identify exon deletions and duplications in the genes *ACVRL1*, *BMPR2* and *ENG* (P093-C2, MRC-Holland, Amsterdam, The Netherlands). Familial variants were sought by Sanger sequencing (ABI Genetic Analyzer 3130xl, Applied Biosystems, MA, USA) or MLPA. Variants were characterized following the American College of Medical Genetics and Genomics guidelines [12]. 

### 2.4. Expression Level Analyses

For each patient, whole blood was collected in PAXgene Blood RNA tubes (bd, Heidelberg, Germany) and serum using serum gel tubes (Sarstedt, Nümbrecht, Germany). RNA was extracted with a PAXgene Blood RNA kit (Qiagen, Hilden, Germany) by a semi-automated procedure following the manufacturer’s protocol (QIAcube, Qiagen, Germany). The extracted RNA was stored at −80 °C before the production of complementary DNA (cDNA) with Transcriptor First Strand cDNA Synthesis Kit (Roche, Basel, Switzerland). Quantitative polymerase chain reaction (qPCR) was performed in accordance with MIQE-guidelines (minimum information for publication of quantitative real-time PCR experiments) using a LightCycler^®^ 480 Real-Time PCR instrument (software version 1.5, Roche, Basel, Switzerland) [13]. Gene-specific primers and probes (Universal ProbeLibrary, Roche, Basel, Switzerland) were used in combination with qPCR Probe-MasterMix. To calculate the difference of the cycle threshold (∆CT) values of *BMPR2*, the CT value of the two housekeeping genes *ESD* (esterase D) and *RPS18* (40S ribosomal protein S18) were considered, following the 2nd derivative maximum method. The detailed procedure is described elsewhere [13]. To better illustrate the values determined, 1/∆CT of the relative *BMPR2* mRNA expression was used in the statistical analysis.

Serum samples were used for enzyme-linked immunosorbent assay (ELISA). Briefly, serum collected from whole blood was frozen at −80 °C to enable a joined analysis of samples and minimize batch effects. Serum from each proband was pipetted into a 96-well plate and treated with ELISA reagents according to the manufacturer’s instructions (human *BMPR2* ELISA kit, Elabscience, Wuhan, China). Results were quantified at 450 nm using a microplate reader (Sunrise, Tecan, Männedorf, Switzerland). For each patient, three technical replicates were measured in ELISA and qPCR.

### 2.5. Statistical Methods

Data including patient characteristics, clinical parameters and laboratory data were evaluated by two professional statisticians (N.B. and S.L.), using descriptive statistics with mean ± standard deviation and frequency tables. Frequency data were presented as n and % and were analyzed using the chi-square test. Clinical characteristics and laboratory data of *BMPR2* variant carriers, non-carriers and healthy controls were compared with Mann–Whitney U test for independent samples. Differences between variant carriers and non-carriers were analyzed by the Mann–Whitney U test and students’ *t*-test. Association between clinical characteristics and laboratory data was tested by Pearson correlation. Parameters with more than 30% missing values were not considered for correlation analysis. *p* values < 0.05 were considered statistically significant. All analyses were performed with SPSS V 25.0 (IBM Corp., Armonk, NY, USA) or SAS 9.4 for Windows (SAS Corp., Charlotte, NC, USA). 

## 3. Results

### 3.1. Baseline Characteristics

**Figure 1:**
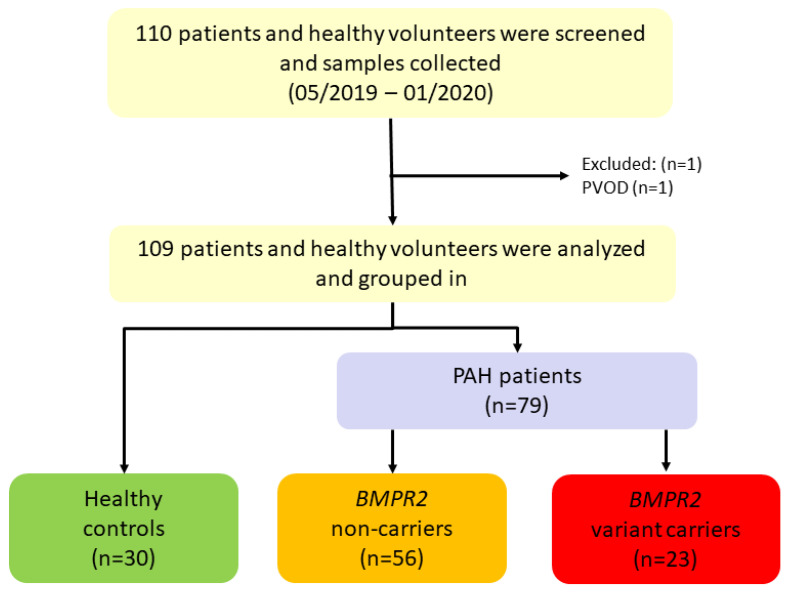
Recruitment of study population from May 2019 to January 2020. 110 participants were screened, and samples were collected of which 109 were analyzed and included. *BMPR2*: bone morphogenetic protein receptor type II, PAH: pulmonary arterial hypertension, PVOD: pulmonary veno-occlusive disease.

Between May 2019 and January 2020, 110 PAH patients and healthy volunteers were screened for this study. One participant was excluded with the differential diagnosis of pulmonary veno-occlusive disease (PVOD). Thus, 79 I/HPAH patients and 30 healthy controls were included (Table 1, Figure 1). Genetic testing revealed 23 of the 79 PAH patients (29.1%) to be *BMPR2* variant-carriers (Figure 1). In the remaining 56 PAH patients (70.9%), no (likely) pathogenic *BMPR2* variant was identified. Of note, two IPAH patients without a disease-causing *BMPR2* variant had a pathogenic missense variant in the gene *SMAD9* and *GDF2*, respectively. The 56 patients were classified as *BMPR2* non-carriers (Figure 1). For all patients and the healthy controls, a *BMPR2* gene mRNA and protein expression analysis was performed in addition to the routine clinical examinations. Out of 79 PAH patients, 58 were female (73%, Table 1). Mean age was 51.4 ± 16 years.

Table 1 shows baseline characteristics of the whole study cohort and of *BMPR2* variant carriers and non-carriers. Right heart catheterization revealed on average severely increased pulmonary pressures with high pulmonary vascular resistance. Most patients showed an enlarged right heart with mild impairment of right ventricular systolic function. On average, patients displayed a normal renal function. One patient, who was a positive vasoresponder, received only calcium channel blocker therapy. Most patients received double or triple combination treatment (Table 1). Healthy controls were age and gender matched to non-variant carriers. Out of 30 healthy controls, 22 were female (73%). The mean age was 46.8 ± 13.9 years (vs. 73% female in the patient cohort with an age of 51.4 ± 16 years).

### 3.2. Clinical Differences between BMPR2 Variant Carriers and Non-Carriers

Patients with a pathogenic *BMPR2* variant suffered from more severe PAH than non-carriers (Table 1). They showed a significantly higher systolic and mean pulmonary arterial pressure (*p* = 0.001 and 0.029, respectively), a significantly higher pulmonary vascular resistance (*p* < 0.001), lower cardiac output (*p* = 0.003) and lower cardiac index (*p* = 0.005) measured by right heart catheterization (Table 1).

### 3.3. BMPR2 Characterization and Expression

The pathogenic variants in the *BMPR2* gene in the 23 variant carriers were characterized as base pair deletions (n = 7) or duplications (n = 2) leading to a frameshift, whole exon deletions (n = 2), whole exon duplications (n = 1), splice site variants (n = 6) or nonsense (n = 5) variants. 

**Figure 2:**
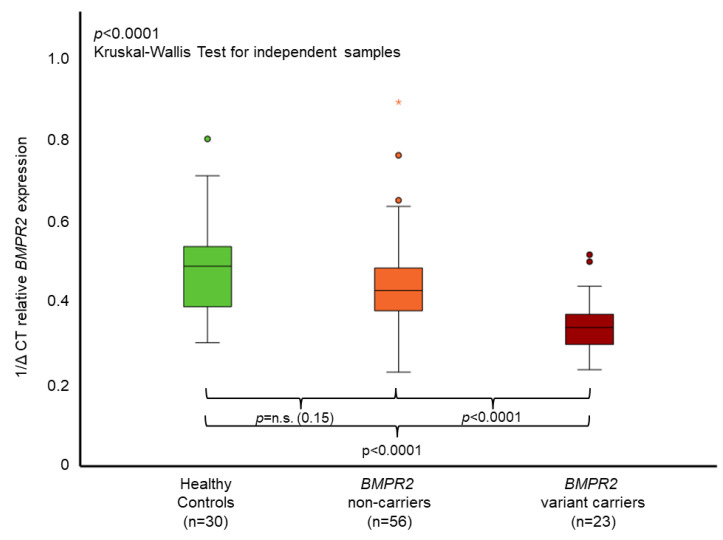
Mean relative *BMPR2* mRNA expression in whole blood. The boxplots provide median values (horizontal lines), interquartile range (box), 1.5× interquartile range (whiskers), outliers (indicated by circles within 1.5 to 3× interquartile range) and extreme outliers (indicated by asterisks > 3× interquartile range). A significant difference of *BMPR2* mRNA expression could be identified between healthy controls, *BMPR2* non-carriers and *BMPR2* variant carriers. With Bonferroni correction, *p*-values were healthy controls vs. non-carriers *p* = 0.453, non-carriers vs. variant carriers *p* = 0.0002, and healthy controls vs. variant carriers *p* < 0.0001. The 1/delta cycle threshold (1/∆CT) denotes the level of *BMPR2* mRNA gene expression measured by qPCR. n.s. = non-significant.

*BMPR2* mRNA expression levels in whole blood measured by qPCR significantly differed between *BMPR2* variant carriers, non-carriers and healthy controls (*p* < 0.0001, Figure 2). *BMPR2* variant carriers had the lowest expression levels. Non-carriers showed lower *BMPR2* mRNA expression in comparison to healthy controls, which was not statistically significant (*p* = 0.151, Figure 2). The two IPAH patients with pathogenic variants in the genes *GDF2* or *SMAD9* had lower *BMPR2* mRNA expression levels (0.394 and 0.433, respectively) than average in the *BMPR2* non-carrier group (0.445), albeit having higher values than the average of the *BMPR2* variant carrier group (0.353).

No significant difference could be identified between the groups on *BMPR2* protein level measured by enzyme-linked immunosorbent assay (ELISA) in sera (healthy controls: 0.31 ± 0.23 ng/mL, non-carriers 0.48 ± 0.43 ng/mL, *BMPR2* variant carriers 0.51 ± 0.49 ng/mL, *p* = 0.52).

### 3.4. Correlation of BMPR2 mRNA Expression with Further Laboratory and Clinical Characteristics

Low *BMPR2* mRNA expression correlated weakly but significantly with low glomerular filtration rate, high hematocrit and high urea levels (Table 2). Patients with lower *BMPR2* mRNA expression had significantly higher systolic pulmonary arterial pressures (sPAP) measured by right heart catheterization and echocardiography, greater right ventricular wall thickness, higher pulmonary vascular resistance and a lower cardiac index and a shorter 6 min walking distance (Table 2).

## 4. Discussion

This study showed that *BMPR2* mRNA levels measured in peripheral blood were significantly reduced in PAH patients with a (likely) pathogenic *BMPR2* variant compared to healthy controls. Non-variant carriers showed a trend of reduced *BMPR2* mRNA expression levels. Furthermore, the study revealed for the first time a significant correlation between lower *BMPR2* mRNA expression levels measured in blood cells and worse values for hemodynamic and laboratory parameters. In particular, the significant correlation of low *BMPR2* mRNA expression with worse hemodynamics and lower exercise capacity suggests that this analysis could be used as a marker for disease severity reflecting underlying pathomechanisms.

### 4.1. Correlation of BMPR2 mRNA Expression with Further Laboratory and Clinical Characteristics

Disease-causing *BMPR2* germline variants seem to reduce mRNA expression not only in pulmonary endothelial cells [6], but also in peripheral blood cells. The two patients with pathogenic variants in *GDF2*, which encodes the *BMPR2* ligand BMP9, and *SMAD9*, which encodes the downstream pathway signaling protein SMAD8, had intermediate *BMPR2* mRNA expression levels compared to the group averages. Thus, also pathogenic variants in other members of the *BMPR2* pathway may be able to reduce *BMPR2* mRNA expression possibly via impaired positive feedback loops.

In our study, a reduced *BMPR2* mRNA expression was also observed in trend in *BMPR2* non-carriers suffering from PAH in comparison to healthy controls, suggesting that there could be different pathomechanisms reducing *BMPR2* mRNA expression apart from pathogenic *BMPR2* variants [6]. These findings could be important for better understanding of the development of PAH not only in variant carriers, but also in PAH patients without *BMPR2* variants. However, a study with larger group sizes is required to draw definite conclusions, as no clear significant differences could be identified in this study, providing only first preliminary data. In a small cohort of 23 PAH patients with mixed etiologies, a reduced *BMPR2* mRNA expression in mononuclear blood cells was previously reported by Spiekerkoetter et al. 2017 in comparison to 13 healthy controls. The same phase IIa study showed a dose-dependent, albeit non-significant, increase in *BMPR2* mRNA expression by the immunomodulator FK506 (tacrolimus), highlighting *BMPR2* as a potential therapeutic target [14].

A recent phase II study demonstrated the TGF-β superfamily ligand trap sotatercept to be a therapeutic option to rebalance growth-promoting and growth-inhibiting signaling in PAH patients [15]. Sotatercept additionally given to a background therapy could significantly decrease pulmonary vascular resistance [15]. The effect of sotatercept on *BMPR2* mRNA expression was not reported. An assessment of *BMPR2* mRNA expression levels in peripheral blood may also prove to be a valuable marker for therapy response in PAH patients.

### 4.2. BMPR2 mRNA Expression and Correlation to Other Parameters

To our knowledge, this is the first study that found a correlation between clinical and laboratory parameters and the *BMPR2* mRNA expression measured in blood cells. The negative correlation to hemodynamic parameters, such as sPAP, pulmonary vascular resistance or right ventricular wall thickness, and the positive correlation with the cardiac index might highlight *BMPR2* mRNA expression to be a potential prognostic factor or indicator of hemodynamic status. Similarly, the positive correlation with 6 min walking distance could highlight an important relationship with exercise capacity. However, future trials are needed to verify these results and to assess if *BMPR2* mRNA expression levels may be a useful marker for follow-up in affected patients and/or for screening in asymptomatic *BMPR2* variant carriers to possibly monitor disease onset. Moreover, further studies are needed to investigate the consistency of *BMPR2* measurements in the same individual or its alterations, e.g., due to clinical deterioration. Similarly, the response in particular to novel, upcoming treatment options addressing the *BMPR2* pathway, such as sotatercept, could be investigated by measuring the effect on *BMPR2* mRNA expression in blood.

Apart from the *BMPR2* gene and its pathway, various other genes have shown to be involved in the pathogenesis of PAH, such as the potassium channel gene *KCNK3* or the transcription factors *SOX17* and *TBX4* [16]. On a molecular level, an array of different pathways contributes to PAH development, such as pathways involved in inflammation, vascular stiffness, platelet-derived growth factor signaling, iron homeostasis or estrogen signaling [17]. Hence, it is also important to consider other factors in future studies and view PAH as a consequence of a multitude of molecular changes.

### 4.3. Limitations

This study did not enroll asymptomatic *BMPR2* variant carriers due to the restricted time frame. Therefore, we will evaluate the differences in *BMPR2* expression in PAH variant carriers and not affected carriers in a subsequent, larger, prospective study. Furthermore, the sample size was not large enough to investigate expression level differences between patients with different types of pathogenetic variants, such as nonsense or frameshift variants. Protein *BMPR2* expression showed no significantly different levels in the serum of our patients. This could also be due to the nature of *BMPR2* being a transmembrane protein, which should be present at lower levels in serum. Finally, the study only included a single time point of measurement. Further studies are required for longitudinal measurements not only to provide a range for intra-patient measurements in stable patients, but also to identify *BMPR2* mRNA expression changes upon treatment changes or clinical worsening.

## 5. Conclusions

This study showed that *BMPR2* mRNA expression levels could be quantified in peripheral blood and were significantly reduced in *BMPR2* variant carriers. Non-carriers showed, in trend, reduced expression rates. Reduced *BMPR2* mRNA expression levels were significantly associated with disease severity. Further studies are needed to evaluate whether *BMPR2* mRNA expression in peripheral blood is useful as a new marker for pathogenesis and disease severity. This study underlines the central role of *BMPR2* signaling in PAH and provides impetus for future research.

## Figures and Tables

**Table 1:** Baseline characteristics of PAH patients.

		All Patients Mean ± SD or n and (%) n = 79	n ^#^	*BMPR2* Non-Carriers Mean ± SD or n and (%) n = 56	n ^#^	*BMPR2* Variant-Carriers Mean ± SD or n and (%) n = 23	n ^#^	*p*-Value °
**Characteristics**								
	Age, years	51.4 ± 16.0		51.7 ± 16.6		50.9 ± 14.7		0.90
	Height, cm	168.5 ± 8.8	78	168.3 ± 9.1	55	169.0 ± 8.2		0.86
	Weight, kg	74.0 ± 16.8	78	73.8 ± 18.0	55	74.5 ± 13.8		0.57
	Body mass index, kg/m^2^	26.0 ± 5.6	78	26.0 ± 6.0	55	26.0 ± 4.4		0.57
	Sex, female	58 (73.4)		41 (73.2)		17 (73.9)		
	Age at diagnosis, years	42.0 ± 23.5	76	45.9 ± 17.8		31.0 ± 33.1	20	0.09
**Laboratory**								
	CRP, mg/L	7.01 ± 9.08	76	6.61 ± 7.25		8.15 ± 13.11	20	0.43
	Hematocrit, L/L	0.42 ± 0.04		0.42 ± 0.04		0.44 ± 0.04		0.11
	Interleukin-6, pg/mL	2.59 ± 3.00	71	2.76 ± 3.39	55	2.01 ± 0.05	16	0.07
	Creatinine, mg/dL	1.12 ± 2.50		1.23 ± 2.97		0.84 ± 0.21		0.66
	Urea, mg/dL	31.7 ± 13.0	78	32.0 ± 15.0		31.0 ± 5.9	22	0.58
	Glomerular filtration rate, %	91.7 ± 26.0		92.9 ± 27.0		88.9 ± 23.7		0.26
	NT-proBNP, ng/L	593.3 ± 1003.2	78	541.2 ± 961.6		725.9 ± 1114.6	22	0.45
**Diffusion capacity of the lung for carbon monoxide, %**		61.1 ± 17.6	78	58.1 ± 18.9	55	68.3 ± 11.1		0.019 *
**6 min walking distance, m**		460.4 ± 112.8	78	449.8 ± 121.1	55	485.7 ± 87.2		0.185
**Echocardiography**								
	Right atrial area, cm^2^	17.2 ± 7.6	78	16.1 ± 6.3		20.1 ± 9.8	221	0.154
	Right ventricular area, cm^2^	19.0 ± 7.0	72	17.9 ± 6.0		22.8 ± 9.1	16	0.022 *
	Systolic pulmonary arterial pressure, mmHg	53.9 ± 27.0		48.0 ± 21.7		68.2 ± 34.1		0.001 *
	Tricuspid annular plane systolic excursion, cm	3.30 ± 3.90	77	2.35 ± 0.49		2.02 ± 0.36	21	0.014 *
**Right heart catheter at diagnosis**								
	Mean pulmonary arterial pressure, mmHg	50.7 ± 14.8	74	48.4 ± 14.4	53	56.7 ± 14.6	21	0.029 *
	Cardiac output, L/min	4.3 ± 1.2	55	4.6 ± 1.2	39	3.6 ± 0.9	16	0.003 *
	Pulmonary arterial wedge pressure, mmHg	9.2 ± 3.4	66	9.3 ± 3.5	50	8.8 ± 3.1	16	0.65
	Pulmonary vascular resistance, WU	10.6 ± 7.1	58	9.1 ± 7.0	41	14.7 ± 6.3	17	<0.001 *
	Cardiac index, L/min/m^2^	2.3 ± 0.6	50	2.5 ± 0.6	39	2.0 ± 0.4	16	0.005 *
**PAH medication ^+^**								
	Calcium channel blocker	1 (1.3)		1 (1.8)		0 (0)		
	Mono therapy	12 (15.2)		11 (19.6)		1 (4.4)		
	Double therapy	39 (49.4)		28 (50.0)		11 (47.8)		
	Triple therapy	27 (34.2)		16 (28.6)		11 (47.8)		

^#^ in case of missing values, n is provided; CRP: C-reactive protein, NT-proBNP: N-terminal prohormone of brain natriuretic peptide, PAH: pulmonary arterial hypertension, WU: wood units. ° comparing *BMPR2* variant carriers and non-carriers; * indicates statistical significance with *p* < 0.05; ^+^ Monotherapy was either therapy with an endothelin receptor antagonist (ERA) or phosphodiesterase 5 inhibitor (PDE5i)/soluble guanylate cyclase stimulator (sGCs); double therapy, a combination of ERA and PDE5i or sGCs; triple therapy, additional prostacyclin analogue or prostacyclin receptor antagonist.

**Table 2:** Correlation of *BMPR2* mRNA expression with laboratory and clinical characteristics.

	1/ΔCT Relative *BMPR2* Expression		
	Pearson Correlation Coefficient	*p*-Value	n ^#^
**Laboratory**			
Hematocrit	−0.242	0.031 *	
Urea, mg/dL	−0.242	0.033 *	78
Glomerular filtration rate, %	0.329	0.003 *	
Creatinine, mg/dL	0.092	0.420	
NT-proBNP, ng/L	−0.204	0.073	78
**Right heart catheterization**			
Systolic pulmonary artery pressure (PAP), mmHg	−0.269	0.031 *	65
Diastolic PAP, mmHg	−0.118	0.349	65
Mean PAP, mmHg	−0.192	0.102	74
Pulmonary artery wedge pressure, mmHg	−0.047	0.710	66
Cardiac output, L/min	0.216	0.113	55
Pulmonary vascular resistance, WU	−0.278	0.035 *	58
Cardiac index, L/min/m^2^	0.285	0.035 *	55
**Transthoracic echocardiography**			
Right ventricular wall thickness	−0.282	0.014 *	76
TAPSE, cm	−0.151	0.191	77
Right atrial area, cm^2^	−0.162	0.156	78
Right ventricular area, cm^2^	−0.169	0.157	72
Systolic PAP, mmHg	−0.271	0.016 *	
**Exercise status**			
6 min walking distance, m	0.344	0.002 *	78

^#^ in case of missing values, n is provided; NT-proBNP: N-terminal prohormone of brain natriuretic peptide, PAP: pulmonary artery pressure, TAPSE: tricuspid annular plane systolic excursion, WU: wood units; * indicates statistical significance with *p* < 0.05.

## Data Availability

The data presented in this study are available in the article.

## References

[1] Galiè N., Humbert M., Vachiery J.L., Gibbs S., Lang I., Torbicki A., Simonneau G., Peacock A., Vonk Noordegraaf A., Beghetti M. (2015). 2015 ESC/ERS Guidelines for the diagnosis and treatment of pulmonary hypertension: The Joint Task Force for the Diagnosis and Treatment of Pulmonary Hypertension of the European Society of Cardiology (ESC) and the European Respiratory Society (ERS): Endorsed by: Association for European Paediatric and Congenital Cardiology (AEPC), International Society for Heart and Lung Transplantation (ISHLT). Eur. Respir. J..

[2] Song J., Eichstaedt C.A., Rodríguez Viales R., Benjamin N., Harutyunova S., Fischer C., Grünig E., Hinderhofer K. (2016). Identification of genetic defects in pulmonary arterial hypertension by a new gene panel diagnostic tool. Clin. Sci..

[3] Machado R.D., Eickelberg O., Elliott C.G., Geraci M.W., Hanaoka M., Loyd J.E., Newman J.H., Phillips J.A., Soubrier F., Trembath R.C. (2009). Genetics and genomics of pulmonary arterial hypertension. J. Am. Coll. Cardiol..

[4] Pfarr N., Szamalek-Hoegel J., Fischer C., Hinderhofer K., Nagel C., Ehlken N., Tiede H., Olschewski H., Reichenberger F., Ghofrani A.H. (2011). Hemodynamic and clinical onset in patients with hereditary pulmonary arterial hypertension and *BMPR2* mutations. Respir. Res..

[5] Eichstaedt C.A., Song J., Benjamin N., Harutyunova S., Fischer C., Grünig E., Hinderhofer K. (2016). EIF2AK4 mutation as “second hit” in hereditary pulmonary arterial hypertension. Respir. Res..

[6] Atkinson C., Stewart S., Upton P.D., Machado R., Thomson J.R., Trembath R.C., Morrell N.W. (2002). Primary pulmonary hypertension is associated with reduced pulmonary vascular expression of type II bone morphogenetic protein receptor. Circulation.

[7] Jonigk D., Golpon H., Bockmeyer C.L., Maegel L., Hoeper M.M., Gottlieb J., Nickel N., Hussein K., Maus U., Lehmann U. (2011). Plexiform lesions in pulmonary arterial hypertension composition, architecture, and microenvironment. Am. J. Pathol..

[8] Evans J.D.W., Girerd B., Montani D., Wang X.-J., Galiè N., Austin E.D., Elliott G., Asano K., Grünig E., Yan Y. (2016). *BMPR2* mutations and survival in pulmonary arterial hypertension: An individual participant data meta-analysis. Lancet Respir. Med..

[9] Hinderhofer K., Fischer C., Pfarr N., Szamalek-Hoegel J., Lichtblau M., Nagel C., Egenlauf B., Ehlken N., Grünig E. (2014). Identification of a new intronic *BMPR2*-mutation and early diagnosis of heritable pulmonary arterial hypertension in a large family with mean clinical follow-up of 12 years. PLoS ONE.

[10] Montani D., Girerd B., Jaïs X., Laveneziana P., Lau E.M.T., Bouchachi A., Hascoët S., Günther S., Godinas L., Parent F. (2021). Screening for pulmonary arterial hypertension in adults carrying a *BMPR2* mutation. Eur. Respir. J..

[11] Eichstaedt C.A., Verweyen J., Halank M., Benjamin N., Fischer C., Mayer E., Guth S., Wiedenroth C.B., Egenlauf B., Harutyunova S. (2020). Myeloproliferative Diseases as Possible Risk Factor for Development of Chronic Thromboembolic Pulmonary Hypertension-A Genetic Study. Int. J. Mol. Sci..

[12] Richards S., Aziz N., Bale S., Bick D., Das S., Gastier-Foster J., Grody W.W., Hegde M., Lyon E., Spector E. (2015). Standards and guidelines for the interpretation of sequence variants: A joint consensus recommendation of the American College of Medical Genetics and Genomics and the Association for Molecular Pathology. Genet. Med..

[13] Schneider M.A., Granzow M., Warth A., Schnabel P.A., Thomas M., Herth F.J., Dienemann H., Muley T., Meister M. (2015). Glycodelin: A New Biomarker with Immunomodulatory Functions in Non-Small Cell Lung Cancer. Clin. Cancer Res..

[14] Spiekerkoetter E., Sung Y.K., Sudheendra D., Scott V., Del Rosario P., Bill M., Haddad F., Long-Boyle J., Hedlin H., Zamanian R.T. (2017). Randomised placebo-controlled safety and tolerability trial of FK506 (tacrolimus) for pulmonary arterial hypertension. Eur. Respir. J..

[15] Humbert M., McLaughlin V., Gibbs J.S.R., Gomberg-Maitland M., Hoeper M.M., Preston I.R., Souza R., Waxman A., Escribano Subias P., Feldman J. (2021). Sotatercept for the Treatment of Pulmonary Arterial Hypertension. N. Engl. J. Med..

[16] Eichstaedt C.A., Sassmannshausen Z., Shaukat M., Cao D., Xanthouli P., Gall H., Sommer N., Ghofrani H.A., Seyfarth H.J., Lerche M. (2022). Gene panel diagnostics reveals new pathogenic variants in pulmonary arterial hypertension. Respir. Res..

[17] Hemnes A.R., Humbert M. (2017). Pathobiology of pulmonary arterial hypertension: Understanding the roads less travelled. Eur. Respir. Rev..

